# Phase 1 trial of olaratumab monotherapy and in combination with chemotherapy in pediatric patients with relapsed/refractory solid and central nervous system tumors

**DOI:** 10.1002/cam4.3658

**Published:** 2021-01-20

**Authors:** Leo Mascarenhas, Chitose Ogawa, Theodore W. Laetsch, Brenda J. Weigel, Michael W. Bishop, Julie Krystal, Scott C. Borinstein, Emily K. Slotkin, Jodi A. Muscal, Pooja Hingorani, Donna E. Levy, Gary Mo, Ashwin Shahir, Jennifer Wright, Steven G. DuBois

**Affiliations:** ^1^ Cancer and Blood Disease Institute Children's Hospital Los Angeles Norris Comprehensive Cancer Center Keck School of Medicine University of Southern California Los Angeles CA USA; ^2^ Department of Pediatric Oncology National Cancer Center Hospital Tokyo Japan; ^3^ Department of Pediatrics Harold C. Simmons Comprehensive Cancer Center UT Southwestern Medical Center Dallas TX USA; ^4^ Pauline Allen Gill Center for Cancer and Blood Disorders Children's Health Dallas TX USA; ^5^ Pediatric Hematology/Oncology University of Minnesota Masonic Cancer Center Minneapolis MN USA; ^6^ Department of Oncology St. Jude Children's Research Hospital Memphis TN USA; ^7^ Department of Pediatric Hematology/Oncology Cohen Children’s Medical Center New Hyde Park NY USA; ^8^ Division of Pediatric Hematology/Oncology Vanderbilt University Medical Center Nashville TN USA; ^9^ Memorial Sloan Kettering Cancer Center New York NY USA; ^10^ Texas Children's Cancer Center Baylor College of Medicine Houston TX USA; ^11^ The University of Texas MD Anderson Cancer Center Houston TX USA; ^12^ Biostatistics and Biometrics Division Syneos Health Morrisville NC USA; ^13^ PK/PD and Pharmacometrics Division Eli Lilly and Company Indianapolis IN USA; ^14^ Oncology Division Eli Lilly and Company Indianapolis IN USA; ^15^ Dana‐Farber/Boston Children’s Cancer and Blood Disorders Center and Harvard Medical School Boston MA USA; ^16^ Department of Pediatrics Harvard Medical School Boston MA USA

**Keywords:** chemotherapy, olaratumab, pediatric cancer, platelet‐derived growth factor receptor

## Abstract

Olaratumab is a monoclonal antibody that specifically binds to platelet‐derived growth factor receptor alpha (PDGFRα) and blocks receptor activation. We conducted a phase 1 trial to evaluate the safety of olaratumab and determine a recommended dose in combination with three different chemotherapy regimens in children. Patients <18 years with relapsed/refractory solid or central nervous system tumors were enrolled to two dose levels of olaratumab. Patients received olaratumab monotherapy at 15 mg/kg (Part A) or 20 mg/kg (Part B) on Days 1 and 8 of the first 21‐day cycle, followed by olaratumab combined with standard fixed doses of chemotherapy with doxorubicin, vincristine/irinotecan, or high‐dose ifosfamide by investigator choice for subsequent 21‐day cycles. In Part C, patients received olaratumab 20 mg/kg plus assigned chemotherapy for all cycles. Parts A‐C enrolled 68 patients across three chemotherapy treatment arms; olaratumab in combination with doxorubicin (N = 16), vincristine/irinotecan (N = 26), or ifosfamide (N = 26). Three dose‐limiting toxicities (DLTs) occurred during olaratumab monotherapy (at 15 mg/kg, grade [G] 4 alanine aminotransferase [ALT]; at 20 mg/kg, G3 lung infection and G3 gamma‐glutamyl transferase). One DLT occurred during vincristine/irinotecan with olaratumab 20 mg/kg therapy (G3 ALT). Treatment‐emergent adverse events ≥G3 in >25% of patients included neutropenia, anemia, leukopenia, lymphopenia, and thrombocytopenia. Pharmacokinetic profiles of olaratumab with chemotherapy were within the projected range based on adult data. There was one complete response (rhabdomyosarcoma [Part B vincristine/irinotecan arm]) and three partial responses (two rhabdomyosarcoma [Part A doxorubicin arm and Part C doxorubicin arm]; one pineoblastoma [Part B vincristine/irinotecan arm]). Olaratumab was tolerable and safely administered in combination with chemotherapy regimens commonly used in children and adolescents.

## Graphical abstract

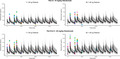

## 1 INTRODUCTION

The platelet‐derived growth factor receptor (PDGFR) pathway has been implicated in several pediatric tumors such as gliomas and sarcomas including rhabdomyosarcoma, osteosarcoma, and Ewing sarcoma.^1^, ^2^, ^3^, ^4^ In pediatric sarcomas and gliomas, several types of genetic alterations, including gene amplifications, translocations, and activating mutations, result in ligand and/or receptor overexpression. PDGFR gene expression analysis in pediatric rhabdomyosarcoma demonstrated decreased failure‐free survival for patients with tumors that overexpressed either PDGFRα or PDGFRβ mRNA.^5^

Olaratumab is a recombinant human immunoglobulin subclass G1 monoclonal antibody (mAb) that specifically binds to PDGFRα, blocking PDGF‐AA, PDGF‐BB, and PDGF‐CC receptor activation.^6^ Olaratumab has demonstrated anticancer activity in *in vitro* and *in vivo* preclinical models known to be driven by a PDGF‐PDGFRα autocrine loop.^7^

In a randomized phase 2 trial, olaratumab in combination with doxorubicin showed an overall survival benefit in adults with advanced soft tissue sarcoma (STS),^8^ which led to accelerated approval by the U.S. Food and Drug Administration.^9^ This approval in turn stimulated the exploration of olaratumab in combination with standard chemotherapy regimens for patients with sarcomas and pediatric cancers.

The phase 1 study reported here (NCT02677116) is an open‐label study of olaratumab in pediatric patients with refractory or relapsed solid or central nervous system (CNS) tumors. The trial was developed to investigate olaratumab as a single agent and in combination with one of three commonly used pediatric chemotherapy regimens in a single study. Doxorubicin, vincristine/irinotecan, and high‐dose ifosfamide are commonly used in primary therapy or salvage chemotherapy for pediatric CNS and solid tumors such as rhabdomyosarcoma and osteosarcom^10^, ^11^, ^12^ and thus were chosen as the chemotherapy backbone options for the trial. We report the safety, pharmacokinetics (PK), and objective radiographic responses observed with olaratumab monotherapy and with combination therapy.

## 2 MATERIALS AND METHODS

### 2.1 Patients

Eligible patients were <18 years of age and had relapsed or refractory solid or CNS tumors, not amenable to curative treatment and for which chemotherapy with doxorubicin, vincristine/irinotecan, or high‐dose ifosfamide was deemed appropriate by the treating investigator. Patients had measurable and/or nonmeasurable but evaluable disease as defined by the Response Evaluation Criteria In Solid Tumors (RECIST version 1.1),^13^ or by the Response Assessment in Neuro‐Oncology (RANO) criteria for CNS tumors,^14^ and adequate hematologic, organ, and coagulation function ≤2 weeks prior to first dose of the study drug. In addition, patients had a Lansky (<16 years of age)^15^ or Karnofsky (≥16 years of age)^16^ performance score ≥50; were fully recovered from the acute effects of all prior anticancer therapies; were able (patient or patient's parent/guardian) to provide informed consent and comply with study procedures; and, if of childbearing potential, had agreed to use adequate contraception if sexually active prior to study entry and for the duration of study participation. Patients with no prior history of anthracycline exposure were able to enroll on the doxorubicin combination arm and had to have a left ventricular ejection fraction ≥50% or shortening fraction ≥27% at baseline, and a corrected QT interval of <480 msec on screening.

Patients were excluded if they had undergone a bone marrow or solid organ transplant (prior autologous stem cell infusion was allowed), or if they had an uncontrolled intercurrent illness including, but not limited to, an ongoing/active infection requiring parenteral antibiotics, symptomatic congestive heart failure, severe myocardial insufficiency, cardiac arrhythmia, cardiomyopathy, or psychiatric illness/social situation that would limit compliance with study requirements. Patients could not concurrently be enrolled in any other type of medical research judged not to be scientifically or medically compatible with this study or have received an investigational agent or non‐approved use of a drug or device within 21 days of the initial dose of study drug.

This study was conducted in accordance with consensus ethics principles derived from international ethics guidelines, including the Declaration of Helsinki and Council for International Organizations of Medical Sciences International Ethical Guidelines. Informed consent was required by a legal representative of the patient prior to participation in this study. In addition to informed consent obtained by a legal representative, the child provided documented assent, if capable.

### 2.2 Study design

This was a multicenter, dose‐escalation, open‐label phase 1 trial with three distinct parts: Parts A, B, and C (Supplementary Figure S1). Since this was a dose‐escalation phase 1 study, the sample size was not based on power, but on the reporting of dose‐limiting toxicities (DLT), as described. In Part A, patients were treated for one 21‐day cycle of olaratumab monotherapy intravenously (IV) at 15 mg/kg on Day 1 and Day 8. If the patient did not experience a DLT in the first cycle of monotherapy, or meet any other criteria for discontinuation, the patient then received subsequent 21‐day cycles of olaratumab (15 mg/kg) plus one of three standard chemotherapy regimens according to investigator choice: doxorubicin (doxorubicin‐naïve patients only), vincristine‐irinotecan, or high‐dose ifosfamide. Of note, other criteria for discontinuation included the following: enrollment in any other clinical trial; physician, parent/legal guardian, or sponsor decision; Grade 3 or 4 infusion‐related reactions/anaphylaxis; withdrawal of consent; or known clinical or radiographic disease progression. Radiographic imaging for disease assessment was not required after Cycle 1. During Cycle 1 monotherapy, if the DLT rate was >33%, the dose of olaratumab was to be reduced to 10 mg/kg. If the DLT rate was >33% in the initial cycle of one of the combination therapy arms (Cycle 2), appropriate dose de‐escalation of olaratumab to 10 mg/kg for that individual combination therapy arm would occur. Part A was to consist of at least 12 evaluable patients.

Part B was initiated when acceptable safety results and PK data from Part A monotherapy were obtained and at least one of the Part A 15‐mg/kg combination arms had a DLT rate of ≤33%. Only the combination arms in Part A meeting these criteria were to be studied in Part B. Patients in Part B were treated identically to patients in Part A except the dose of olaratumab was 20 mg/kg. Part B was considered complete when at least 10 patients (regardless of assigned chemotherapy arm) were evaluable for safety of olaratumab 20 mg/kg monotherapy. In Part B, if a patient had a DLT during Cycle 1 monotherapy, the subsequent olaratumab dose was to be reduced to 15 mg/kg, and the patient proceeded to combination therapy in Cycle 2. During Cycle 1 monotherapy, if the DLT rate was >33%, the dose of olaratumab was to be reduced to 15 mg/kg for subsequently enrolled patients. If the DLT rate was >33% in any combination therapy arm, appropriate dose de‐escalation of olaratumab to 15 mg/kg for that individual combination therapy arm would occur.

Part C patients received olaratumab (20 mg/kg) in combination with any of the three chemotherapy regimens from Cycle 1 onwards (ie, no olaratumab monotherapy in Cycle 1). If the DLT rate was >33% during Cycle 1 in one of the combination therapy arms, the dose of olaratumab was to be reduced to 15 mg/kg. For all parts, patients continued until disease progression or other discontinuation criteria were met. Parts B and C combined were planned to enroll up to 45 patients (15 perchemotherapyarm).

The primary objective of this study was to determine a recommended dose of olaratumab in combination with at least one of the studied chemotherapy regimens in pediatric patients based on any DLTs as well as olaratumab serum exposure matching between adults and children. The secondary objectives were to investigate the PK of olaratumab as monotherapy and in combination with either doxorubicin, vincristine‐irinotecan, or high‐dose ifosfamide; to assess the possible development of antibodies against olaratumab (immunogenicity); and to document any observed antitumor activity.

### 2.3 Treatment

Olaratumab 15 mg/kg (Part A) and olaratumab 20 mg/kg (Part B and Part C) were administered intravenously over one hour on Days 1 and 8 of each 21‐day cycle. During Cycle 1 (both Day 1 and Day 8), all patients received premedication with dexamethasone (or equivalent) due to infusion‐related reactions (IRR) observed with olaratumab. For all subsequent cycles, all patients received premedication with an H1 antagonist intravenously prior to each dose.

During combination therapy, all chemotherapy agents were administered after olaratumab (Part A and Part B [Cycles 2 + n] and Part C [all cycles]) according to the following schedule: doxorubicin 37.5 mg/m^2^ IV on Days 1 and 2 for up to six cycles or a cumulative dose of 450 mg/m^2^; ifosfamide 2.8 g/m^2^ IV over 2–3 hours on Days 1–5 for up to six cycles or a cumulative dose of 84 gm/m^2^; vincristine 1.5 mg/m^2^ (patients ≥10 kg) or 0.05 mg/kg (patients <10 kg) with maximum dose of 2 mg IV for all patients on Days 1 and 8; and irinotecan 50 mg/m^2^ IV on Days 1–5. Patients were eligible to continue olaratumab monotherapy after completion of chemotherapy until meeting criteria for discontinuation. Myeloid growth factors and dexrazoxane were allowed during combination therapy.

### 2.4 Dose‐limiting toxicities

A DLT was defined as an adverse event (AE) during the first 21 days of monotherapy or combination therapy that was at least possibly related to the study treatment and fulfilled any one of the following criteria using the National Cancer Institute's (NCI) Common Terminology Criteria for Adverse Events (CTCAE) version 4.0: grade ≥3 non‐hematologic toxicity (exceptions were made for the following grade 3 AEs: uncomplicated febrile neutropenia; nausea; vomiting; diarrhea; transient electrolyte abnormalities; constipation that could be controlled within 48 hours; transient elevations of alanine aminotransferase [ALT] and/or aspartate aminotransferase [AST] lasting fewer than 8 days, without evidence of other hepatic injury), grade 4 neutropenia lasting longer than 2 weeks, grade ≥3 thrombocytopenia complicated by hemorrhage; any hematologic toxicity causing a cycle delay of >14 days, and any other significant toxicity deemed by the primary investigator and sponsor to be dose limiting.

### 2.5 Safety and efficacy assessments

Adverse events were coded using Medical Dictionary for Regulatory Activities (MedDRA) version 21.0. Imaging studies and tumor assessments appropriate to each patient were obtained at baseline and then every two cycles (±seven days) until documented progression for patients with complete response (CR), partial response (PR), or stable disease (SD). For patients who discontinued study treatment due to toxicity or reasons other than progressive disease (PD), imaging studies and tumor assessments were obtained every six weeks (±seven days) until progression.

Extent of disease was assessed by investigator report (RECIST v1.1)^13^ except CNS tumors for which investigators could choose to use RANO‐high‐grade glioma (RANO‐HGG)^14^ criteria. To confirm objective responses, the same radiologic method used for the initial response determination was repeated at least six weeks (two cycles) following the initial observation of an objective response. If a patient was discontinued from the study, repeat radiology assessments could be omitted if clear clinical signs of PD were present.

### 2.6 Pharmacokinetic and immunogenicity evaluations

The PK profile of olaratumab was assessed using peak and trough serum concentrations collected in the first three treatment cycles, and trough concentrations in subsequent cycles. Serum samples were analyzed for olaratumab concentration using a validated enzyme‐linked immunosorbent assay method at ICON Laboratory Services, Inc. (Whitesboro, New York, USA). The analytic range of quantification was 1,000 – 100,000 ng/mL. Samples above the limit of quantification were diluted to yield results within the calibrated range.

PK analyses were conducted on patients who received at least one dose of the study drug and had serum samples collected. Data were analyzed using descriptive statistics. Serum olaratumab concentrations from this trial were also overlaid with simulated prediction from a population PK model developed using adult PK data with allometric adjustments to account for the pediatric patient population. Model parameters are provided in the Supporting Information (Supplementary Methods).

Immunogenicity serum samples were collected on Day 1 of each cycle prior to olaratumab infusion and at any time during the 30‐day follow‐up visit. Samples were analyzed for anti‐olaratumab antibodies using a validated immunoassay at Pharmaceutical Product Development (Richmond, Virginia, USA). Samples were assessed using a four‐tiered approach for the detection, confirmation, titer determination, and characterization of neutralizing activity of anti‐olaratumab antibodies. The anti‐olaratumab antibody assay had a minimal required dilution of 1:10, a validated sensitivity of 13.7 ng/mL, and a drug tolerance of >500 µg/mL olaratumab in the presence of 500 ng/mL affinity‐purified hyper‐immunized monkey anti‐olaratumab antibody.

### 2.7 Statistical analyses

Part A and B patients who completed Cycle 1 or discontinued treatment due to an AE during Cycle 1 were considered DLT‐evaluable for olaratumab monotherapy. Part A and B patients who completed Cycle 2 or discontinued treatment due to an AE during Cycle 2 and Part C patients who completed Cycle 1 or discontinued treatment due to an AE during Cycle 1 were considered DLT‐evaluable for combination treatment.

Descriptive statistics were calculated using data from all patients who received any quantity of study drug (safety population) for safety and efficacy outcomes. Safety analyses were conducted with patients grouped according to the actual dose level received.

Exploratory efficacy analyses investigated antitumor activity within each combination arm of the safety population. Progression‐free survival curves and the median with 95% confidence interval (CI) were estimated using the Kaplan‐Meier method.^17^ The objective response rate (ORR = CR + PR) and disease control rate (DCR = CR + PR+ SD) were tabulated for each cohort.

## 3 RESULTS

### 3.1 Patient demographics and disposition

Overall, 68 patients received treatment with olaratumab across the treatment arms in Parts A (N = 30), B (N = 24), and C (N = 14) combined: vincristine/irinotecan (N = 26); doxorubicin (N = 16); or ifosfamide (N = 26). Demographics and disease characteristics for these 68 patients are presented in Table 1. The median age was 11 years (mean, 10; standard deviation [SD], 4.8; range: 2 to 17), 40 patients (59%) were male, and 46 (68%) were white. The median time from initial diagnosis to enrollment was 18 months (mean, 24.5; SD, 24.1; range: 2 to 139). The most common diagnoses were rhabdomyosarcoma (n = 19; 28%) and osteosarcoma (n = 19; 28%). There were 11 (16%) patients treated who had CNS tumors (Table 1).

**Table 1:** Patient demographic characteristics at baseline and pretreatment disease characteristics (safety population)

Characteristics		Number of Patients									
		Part A N = 30			Part B N = 24			Part C N = 14			Total N = 68
		Olaratumab 15 mg with			Olaratumab 20 mg with			Olaratumab 20 mg with			
		Dox n = 11	Vin/Irin n = 10	Ifos n = 9	Dox n = 1	Vin/Irin n = 10	Ifos n = 13	Dox n = 4	Vin/Irin n = 6	Ifos n = 4	
Sex, n (%)											
Male		8 (73)	6 (60)	7 (78)	1 (100)	4 (40)	7 (54)	2 (50)	2 (33)	3 (75)	40 (59)
Female		3 (27)	4 (40)	2 (22)	0	6 (60)	6 (46)	2 (50)	4 (67)	1 (25)	28 (41)
Race, n (%)											
Asian		1 (9)	0	1 (11)	0	0	0	3 (75)	3 (50)	3 (75)	11 (16)
Black or African American		1 (9)	1 (10)	2 (22)	0	2 (20)	2 (15)	0	0	0	8 (12)
White		8 (73)	9 (90)	5 (56)	1 (100)	7 (70)	11 (85)	1 (25)	3 (50)	1 (25)	46 (68)
Missing		1 (9)	0	1 (11)	0	1 (10)	0	0	0	0	3 (4)
Age (years)											
Median (range)		5 (2–15)	10 (2–17)	12 (4–16)	10 (10–10)	12 (3–16)	13 (4–17)	12 (5–15)	10 (2–16)	8 (2–15)	11 (2–17)
Mean (SD)		7 (4)	10 (6)	11 (4)	10 (NA)	11 (5)	13 (4)	11 (5)	10 (5)	8 (7)	10 (5)
Diagnosis, n (%)											
Rhabdomyosarcoma		8 (73)	3 (30)	1 (11)	1 (100)	2 (20)	0	2 (50)	2 (33)	0	19 (28)
Osteosarcoma		0	2 (20)	4 (44)	0	2 (20)	9 (69)	0	0	2 (50)	19 (28)
CNS tumors^a^		0	2 (20)	3 (33)	0	3 (30)	1 (8)	0	2 (33)	0	11 (16)
Other^b^		3 (27)	3 (30)	1 (11)	0	3 (30)	3 (23)	2 (50)	2 (33)	2 (50)	19 (28)
Time from diagnosis to enrollment (months)											
n		9	10	8	1	10	12	4	5	3	62
Median (min‐max)		21 (4–125)	26 (9–50)	14 (2–22)	15 (15–15)	20 (5–139)	16 (6–53)	37 (4–44)	45 (10–54)	13 (8–18)	18 (2–139)
Mean (SD)		28 (38)	26 (15)	12 (8)	15 (NA)	32 (39)	19 (14)	30 (18)	37 (18)	13 (5)	25 (24)
Systemic therapy: number of regimens for locally advanced/metastatic disease, n (%)											
0		7 (64)	3 (30)	5 (56)	1 (100)	7 (70)	9 (69)	1 (25)	1 (17)	1 (25)	35 (51)
1		3 (27)	1 (10)	2 (22)	0	1 (10)	1 (8)	1 (25)	1 (17)	0	10 (15)
2		0	3 (30)	2 (22)	0	1 (10)	0	0	1 (17)	2 (50)	9 (13)
≥3		1 (9)	3 (30)	0	0	1 (10)	3 (23)	2 (50)	3 (50)	1 (25)	14 (21)

Abbreviations: CNS, central nervous system; Dox, doxorubicin; Ifos, ifosfamide; n, number of patients in specified category; N, number of patients per treatment arm; NA, not available; SD, standard deviation; Vin/Irin, vincristine/irinotecan.

^a^ Includes the following CNS tumor types: anaplastic astrocytoma, spinal cord astrocytoma, ependymoma, glioblastoma, medulloblastoma, pineoblastoma, and choroid plexus carcinoma.

^b^ Includes the following tumor types: desmoid tumor, hepatoblastoma, neuroblastoma, mucinous adenocarcinoma, NUT midline carcinoma, sacrococcygeal germ cell tumor, Wilms’ tumor, Ewing sarcoma, and other sarcomas.

### 3.2 Treatment and dose modifications

Median treatment duration (weeks) for Parts A, B, and C, respectively, was: 7 (n = 30; mean, 13; SD, 16), 7 (n = 24; mean, 12; SD, 11), and 13 (n = 14; mean, 15; SD, 10) for olaratumab (all chemotherapy arms combined); 10 (n = 7; mean, 22; SD, 27), 4 (n = 9; mean, 11; SD, 15), and 13 (n = 6; mean, 14; SD, 9) for vincristine/irinotecan; and 4 (n = 9; mean, 9; SD, 7), 4 (n = 11; mean, 9; SD, 8), and 13 (n = 4; mean, 13; SD, 10) for ifosfamide. Median treatment duration for doxorubicin was 7 weeks (n = 6; mean, 10; SD, 8) for Part A and 12 weeks (n = 4; mean, 11; SD, 8) for Part C. For Part B, the one patient enrolled in the olaratumab plus doxorubicin arm completed Cycle 1 only and therefore did not receive any doxorubicin (Supplementary Table S1).

Olaratumab dose modifications or delays occurred in 25 (37%) of 68 patients who received olaratumab across all study parts: 11 (37%) of 30 patients in Part A; eight (33%) of 24 patients in Part B; six (43%) of 14 patients in Part C. Twenty‐six (46%) of the 56 patients who received at least one dose of chemotherapy experienced a dose modification or delay of the chemotherapeutic agent (doxorubicin, n = 1; vincristine, n = 8; irinotecan, n = 6; ifosfamide, n = 11). The majority of these were dose delays due to AEs. Three patients (4.4%) discontinued due to a treatment‐related AE.

### 3.3 Dose‐limiting toxicities

Four patients (6%) had a DLT in Cycle 1. In Part A (olaratumab [15 mg/kg] monotherapy, N = 30), one patient (3%) had grade 4 elevated ALT (confounded by concurrent antibiotic therapy). In Part B (olaratumab [20 mg/kg] monotherapy, N = 24), one patient (4%) had a DLT of grade 3 elevated gamma‐glutamyl transferase, and one patient (4%) had a DLT of grade 3 lung infection. In Part C (olaratumab [20 mg/kg] combination therapy, N = 14), one patient (7%) in the vincristine/irinotecan treatment arm had a DLT of grade 3 elevated ALT. This DLT rate did not meet protocol requirements for dose level de‐escalation. No DLTs occurred in Cycle 2 in any of the three olaratumab combination therapy arms in Part A or B.

### 3.4 Other toxicities

#### 3.4.1 Olaratumab monotherapy

A total of 54 patients received olaratumab monotherapy in Cycle 1 (Part A, N = 30; Part B, N = 24). In Part A, 27 (90%) of 30 patients had ≥1 treatment‐emergent adverse event (TEAE) on olaratumab monotherapy. The most common (incidence ≥3) TEAEs were vomiting (n = 8; 27%), anemia (n = 6; 20%), fatigue, pyrexia, and white blood cell count decreased (n = 4 for each event; 13%), headache, hypertension, hypophosphatemia, insomnia, and nausea (n = 3 for each event; 10%). Of these common TEAEs, all were grade <3, except for two grade 3 anemia events. In Part B, 22 (92%) of 24 patients had ≥1 TEAE on olaratumab monotherapy. The most common (incidence ≥3) TEAEs were headache and nausea (n = 7 for each event; 29%), arthralgia (n = 5; 21%), anemia and decreased appetite (n = 4 for each event; 17%), back pain, constipation, fatigue, hypoalbuminemia, hyponatremia, and pyrexia (n = 3 for each event; 13%). Of these common TEAEs, grade 3 events consisted of headache, anemia, decreased appetite, hypoalbuminemia, and pyrexia in one patient each. Grade 4 TEAEs occurring during olaratumab monotherapy occurred in Part A only and included increased ALT and hypercalcemia (n = 1 for each event; 3%). Of the olaratumab monotherapy TEAEs in Parts A and B, those deemed treatment‐related were mostly grade 1 and 2 and similar to the overall TEAEs described above with the exception that fewer were hematologic (Supplementary Table S2).

Six (11%) of 54 patients who received olaratumab monotherapy had ≥1 serious AE (SAE). Treatment‐related SAEs with olaratumab monotherapy occurred in only one patient in Part A (elevated ALT and AST). Two patients discontinued olaratumab monotherapy due to an AE (grade 3 ascites [Part A] and grade 3 bone pain [Part B]), though neither AE was assessed as related to study treatment.

#### 3.4.2 Olaratumab monotherapy and combination therapy toxicities combined (all cycles)

Table 2 presents an overview of AEs for all patients (N = 68) for all cycles, including olaratumab in combination with doxorubicin, vincristine/irinotecan, or high‐dose ifosfamide in Parts A, B, and C. All patients (100%) experienced ≥1 TEAE, of which 91% were considered treatment‐related. Fifty‐seven (84%) patients had at least one grade ≥3 TEAE, of which 71% were considered treatment‐related. Table 3 presents a summary of any grade TEAEs occurring in >25% of patients, and grade ≥3 TEAEs for all cycles and study parts (A, B, and C) combined. Treatment‐related TEAEs (grade ≥3) are presented in Supplementary Table S3 by study part. Grade ≥3 treatment‐related TEAEs occurring in ≥3 patients in the olaratumab with doxorubicin arms (N = 16) included the following: neutropenia (n = 9; 56%), leukopenia (n = 7; 44%), anemia (n = 4; 25%), and thrombocytopenia and lymphopenia (n = 3 each; 19%); in the olaratumab with vincristine/irinotecan arms (N = 26): neutropenia (n = 10; 38%), anemia (n = 6; 23%), and lymphopenia (n = 4; 15%); and in the olaratumab with ifosfamide arms (N = 26): anemia (n = 15; 58%), thrombocytopenia (n = 14; 54%), leukopenia (n = 13; 50%), neutropenia (n = 11; 42%), and lymphopenia (n = 10; 38%).

**Table 2:** Overview of adverse events for all patients for all cycles across treatment arms in Parts A, B, and C (safety population)

	Part A N = 30			Part B N = 24			Part C N = 14			Total N = 68
	Olaratumab 15 mg with			Olaratumab 20 mg with			Olaratumab 20 mg with			
	Dox N = 11	Vin/Irin N = 10	Ifos N = 9	Dox N = 1	Vin/Irin N = 10	Ifos N = 13	Dox N = 4	Vin/Irin N = 6	Ifos N = 4	
Number of patients who received chemotherapy drug(s)	Dox n = 6	Vin/Irin n = 7	Ifos n = 9	Dox n = 0	Vin/Irin n = 9	Ifos n = 11	Dox n = 4	Vin/Irin n = 6	Ifos n = 4	n = 56
Number of patients with events^a^	n (%)	n (%)	n (%)	n (%)	n (%)	n (%)	n (%)	n (%)	n (%)	n (%)
Patients with ≥1 TEAE	11 (100)	10 (100)	9 (100)	1 (100)	10 (100)	13 (100)	4 (100)	6 (100)	4 (100)	68 (100)
Related to study treatment^b^	7 (64)	10 (100)	9 (100)	1 (100)	9 (90)	12 (92)	4 (100)	6 (100)	4 (100)	62 (91)
Patients with ≥1 grade 3/4 TEAE	8 (73)	7 (70)	9 (100)	1 (100)	8 (80)	10 (77)	4 (100)	6 (100)	4 (100)	57 (84)
Related to study treatment^b^	5 (46)	3 (30)	8 (89)	1 (100)	7 (70)	10 (77)	4 (100)	6 (100)	4 (100)	48 (71)
Patients with ≥1 SAE	2 (18)	3 (30)	4 (44)	0	5 (50)	7 (54)	4 (100)	2 (33)	1 (25)	28 (41)
Related to study treatment^b^	1 (9)	1 (10)	4 (44)	0	3 (30)	6 (46)	2 (50)	1 (17)	1 (25)	19 (28)
Patients discontinued treatment due to AE	1 (9)	0	0	0	0	1 (8)	0	1 (17)	0	3 (4)
Related to study treatment^b^	0	0	0	0	0	0	0	1 (17)	0	1 (2)
Patients discontinued due to SAE	0	0	0	0	0	0	0	0	0	0
Deaths^c^ due to AE while on study treatment	0	0	0	0	0	0	0	0	0	0
Deaths^c^ due to AE within 30 days after treatment discontinuation	0	0	0	0	0	0	0	0	0	0

Abbreviations: AE, adverse event; Dox, doxorubicin; Ifos, ifosfamide; N, number of patients per treatment arm; n, number of patients with specified event; SAE, serious adverse event; TEAE, treatment‐emergent adverse event; Vin/Irin, vincristine/irinotecan.

^a^ Patients may be counted in >1 category.

^b^ Includes events considered related to study treatment, as judged by the investigator.

^c^ Deaths are also included as SAEs and discontinuations due to AEs.

**Table 3:** Treatment‐emergent adverse events by consolidated term occurring in >25% of patients regardless of toxicity, and treatment‐emergent adverse events occurring in ≥2 patients at grade ≥3 toxicity for all cycles across all treatment arms for Parts A, B, and C combined (safety population, N = 68).

TEAEs occurring in >25% of patients regardless of toxicity grade	Any Grade n (%)
Anemia^a^	41 (60)
Leukopenia^b^	36 (53)
Neutropenia^c^	36 (53)
Thrombocytopenia^d^	29 (43)
Musculoskeletal pain^e^	27 (40)
Lymphopenia^f^	24 (35)
Fatigue^g^	19 (28)

TEAEs occurring in ≥2 patients at grade ≥3 toxicity	Grade ≥3 n (%)
Neutropenia^c^	30 (44)
Anemia^a^	29 (43)
Leukopenia^b^	26 (38)
Lymphopenia^f^	19 (28)
Thrombocytopenia^d^	18 (27)
Hypokalemia^h^	5 (7)
Musculoskeletal pain^e^	3 (4)
Mucositis^i^	2 (3)

Abbreviations: n, number of patients in specified category; N, number of patients who received any quantity of study drug; TEAEs, treatment‐emergent adverse events.

^a^ Preferred term reported: anemia.

^b^ Preferred terms reported: decreased white blood cell count, leukopenia.

^c^ Preferred terms reported: decreased neutrophil count, neutropenia.

^d^ Preferred terms reported: decreased platelet count, thrombocytopenia.

^e^ Preferred terms reported: arthralgia, back pain, pain in extremity, musculoskeletal pain, bone pain, musculoskeletal chest pain, myalgia, neck pain.

^f^ Preferred terms reported: decreased lymphocyte count, lymphopenia.

^g^ Preferred term reported: fatigue.

^h^ Preferred term reported: hypokalemia.

^i^ Preferred term reported: stomatitis.

Overall, 28 (41%) of the 68 patients had ≥1 SAE during study treatment, of which 19 (28%) were considered treatment‐related (Supplementary Table S4). The most common treatment‐related SAE was febrile neutropenia (n = 11; 16%). One patient (Part C, Cycle 1) discontinued study treatment during combination therapy due to a treatment‐related AE of grade 2 ALT increased.

#### 3.4.3 Adverse events of special interest

Adverse events of special interest (AESI) included IRRs and cardiac dysfunction or cardiac arrhythmias. Among the 68 patients, eight (12%) had immediate IRRs (defined as occurring the same day as olaratumab infusion) with three patients having an IRR with the first infusion of olaratumab; seven (10%) patients had delayed IRRs. All IRRs were grade 1 or 2 events and no patient with an IRR had treatment‐emergent antidrug antibodies (TEADA). Fourteen (21%) patients had cardiac arrhythmia and seven (10%) patients had cardiac dysfunction AESIs. Of the 15 patients assigned to olaratumab and doxorubicin therapy across the three study parts, one (7%) patient had an AESI of sinus tachycardia, one (7%) patient had electrocardiogram QT corrected interval prolonged, and one (7%) patient had peripheral edema. No patient discontinued treatment due to an AESI.

#### 3.4.4 Pharmacokinetics

A total of 389 serum samples from 67 patients were analyzed for olaratumab concentration.

**FIGURE 1:**
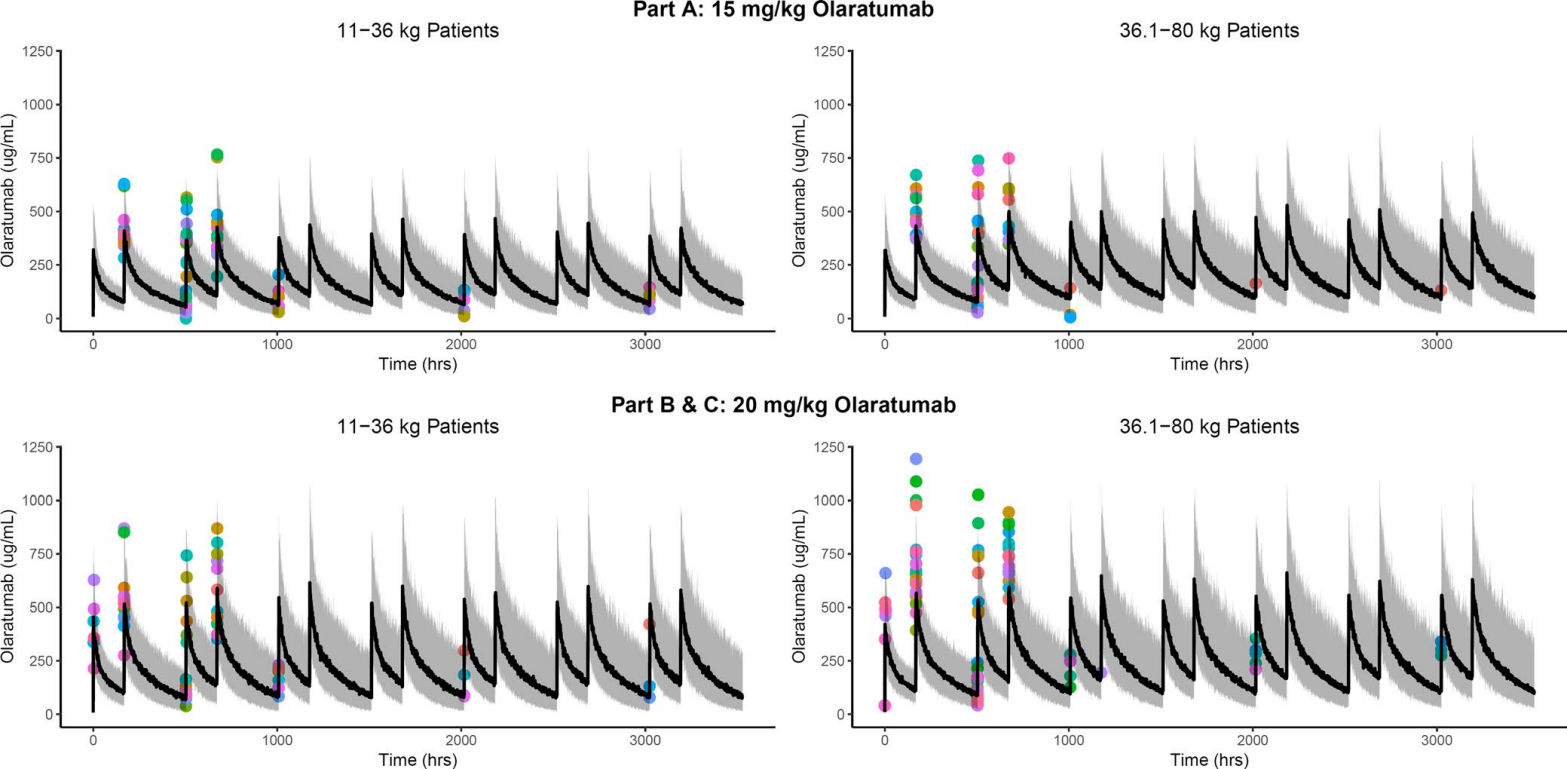
Observed pharmacokinetic (PK) data and model predicted PK profiles by weight in pediatric patients treated with olaratumab. The overlay was limited to the first seven cycles where observed data were available to provide meaningful comparison. Overall, there was general agreement between the observed and model predicted serum concentrations as the observed values (colored circle) overlap with the prediction interval (shaded region) from the PK model. The colored circles correspond to each patient in the study. The shaded region represents the 90% prediction interval, and the solid line represents the median of predicted concentration.

Peak serum geometric mean concentrations ranged from 363 to 707 µg/mL, and trough geometric mean concentrations ranged from 38 to 348 µg/mL. Peak concentrations were higher on Day 8 compared to Day 1, and trough concentrations were also generally higher in later treatment cycles. Patients in Parts B and C had higher serum olaratumab concentrations compared to patients in Part A (Supplementary Figure S2). Serum olaratumab concentrations were similar when administered alone or in combination with chemotherapy (Parts A and B), and similar across the three chemotherapy combinations (Supplementary Figure S2). In order to evaluate the PK characteristics of olaratumab identified in this study in the context of results from prior studies in adults, the observed serum concentration of olaratumab from the current study was overlayed with the predicted population PK model values for pediatric patients derived from adult studies of olaratumab (Figure 1). The concentrations of olaratumab collected in this study were generally within the prediction intervals of the population PK model.

An exploratory analysis did not demonstrate an apparent difference in the rates of TEAEs (any grade or grade ≥3) by serum olaratumab exposure by quartile (Supplementary Table S5).

### 3.5 Immunogenicity

None of the 67 patients with evaluable samples had TEADA. Two patients (3%), both in Part C, had at least one anti‐olaratumab antibody positive result, but neither result met the threshold for TEADA.

### 3.6 Efficacy

Median progression‐free survival (95% CI), best overall response, and overall response rates per treatment arm per study part are presented in Table 4. Overall, 60 (88%) of 68 patients were evaluable for objective response by investigator assessment (RECIST 1.1 or RANO‐HGG criteria). Eight patients were not evaluable (12% of 68): six due to no post‐baseline data, one due to no measurable disease at baseline, and one due to unknown disease status at baseline. One (10%) patient with alveolar rhabdomyosarcoma in Part B vincristine/irinotecan arm had a complete response. Three patients had a partial response: one (9%) patient with alveolar rhabdomyosarcoma in Part A doxorubicin arm, one (10%) patient with pineoblastoma in Part B vincristine/irinotecan arm, and one (25%) patient with rhabdomyosarcoma in Part C doxorubicin arm. Twenty‐one (31%) patients across the three treatment arms for all study parts had SD and 35 (51%) patients had PD.

**Table 4:** Progression‐free survival and best overall response by investigator assessment (RECIST 1.1 or RANO‐HGG criteria; safety population).

	Part A			Part B			Part C		
	Olaratumab 15 mg with			Olaratumab 20 mg with			Olaratumab 20 mg with		
	Dox N = 11	Vin/Irin N = 10	Ifos N = 9	Dox N = 1	Vin/Irin N = 10	Ifos N = 13	Dox N = 4	Vin/Irin N = 6	Ifos N = 4
Progression‐free survival									
Median, months	1	2	1	NE	1	1	6	4	5
95% CI	0, 5	1, 16	1, 6		1, 11	1, 3	1, 6	1, 4	1, 9
Best overall response, n (%)									
Complete response	0	0	0	0	1 (10)	0	0	0	0
Partial response	1 (9)	0	0	0	1 (10)	0	1 (25)	0	0
Stable disease	3 (27)	4 (40)	2 (22)	0	1 (10)	4 (31)	2 (50)	3 (50)	2 (50)
Progressive disease	5 (46)	4 (40)	7 (78)	0	7 (70)	8 (62)	1 (25)	1 (17)	2 (50)
Not evaluable^a^	2 (18)	2 (20)	0	1 (100)	0	1 (8)	0	2 (33)	0
Overall response rate (CR/PR), n (%)	1 (9)	0	0	0	2 (20)	0	1 (25)	0	0
Disease control rate (CR/PR/SD), n (%)	4 (36)	4 (40)	2 (22)	0	3 (30)	4 (31)	3 (75)	3 (50)	2 (50)

Abbreviations: CI, confidence interval; CR, complete response; Dox, doxorubicin; Ifos, ifosfamide; n, number of patients in specified category; N, number of patients per treatment arm; NE, not evaluable; PR, partial response; RANO‐HGG, Response Assessment in Neuro‐Oncology high‐grade glioma; RECIST, Response Evaluation Criteria In Solid Tumors; SD, stable disease; Vin/Irin, vincristine/irinotecan.

^a^ Patients were classified as not evaluable for response per RECIST 1.1 or RANO‐HGG criteria when an incomplete radiologic assessment of target lesions was performed, or there was a change in the method of measurement from baseline that impacted the ability to make a reliable evaluation of response.

**FIGURE 2:**
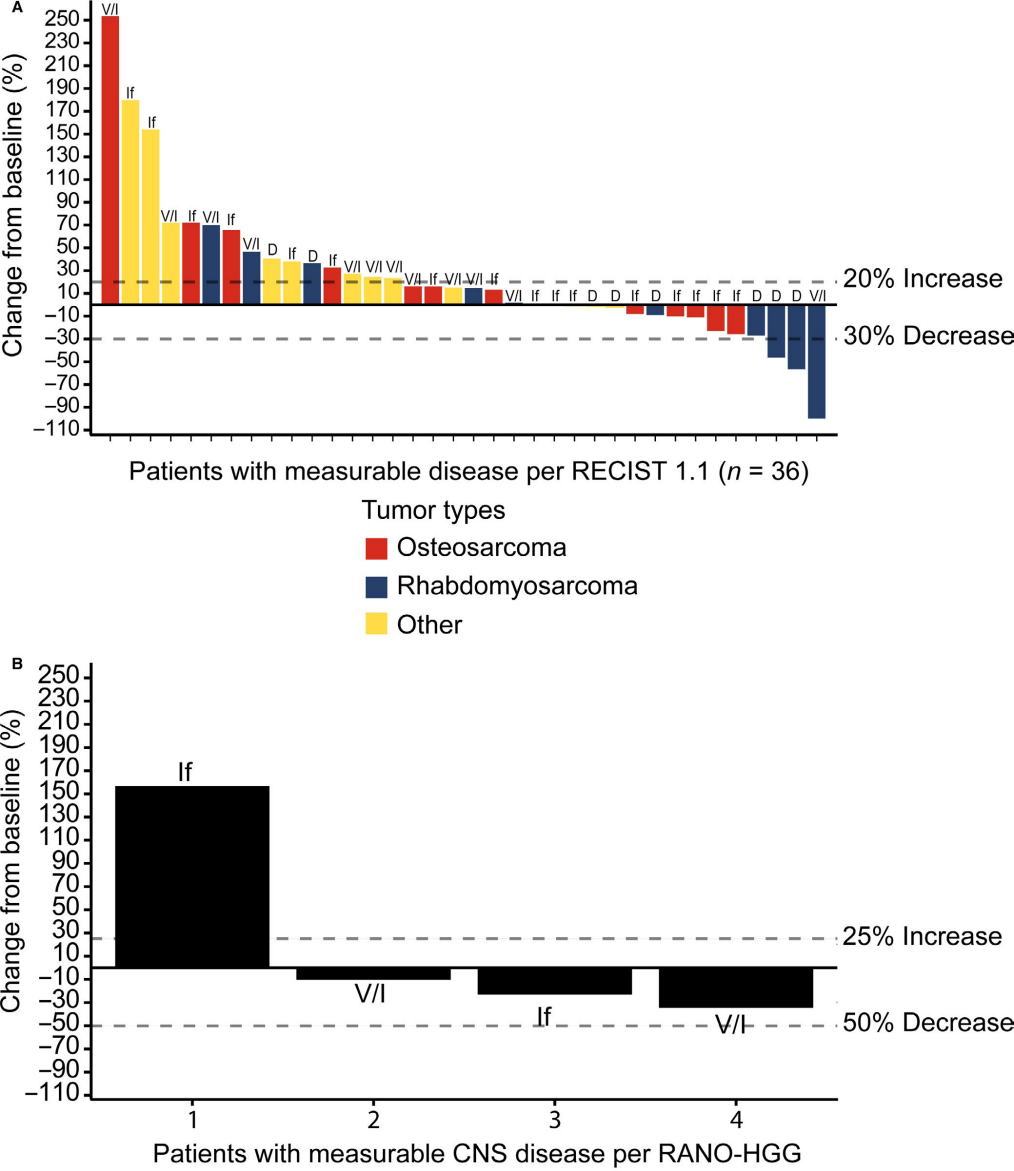
Waterfall plots of best percent change from baseline in tumor size for patients with measurable disease per RECIST 1.1 (**A**) or RANO‐HGG (**B**) criteria. Abbreviations: CNS, central nervous system; D, doxorubicin; If, ifosfamide; RANO‐HGG, Response Assessment in Neuro‐Oncology high‐grade glioma; RECIST, Response Evaluation Criteria In Solid Tumors; V/I, vincristine/irinotecan.

Figure 2 shows the waterfall plot of the best percent change in tumor size from baseline for patients with measurable disease.

## 4 DISCUSSION

The intent of this phase 1 study was to assess olaratumab for the first time in a pediatric population. The primary objective was to determine a recommended dose of olaratumab in combination with either doxorubicin, vincristine/irinotecan, or high‐dose ifosfamide in children based on DLTs and olaratumab serum exposure matching between the adult and pediatric populations. DLT rates during both olaratumab monotherapy and olaratumab combination therapy were low and did not deter dose escalation. No DLTs occurred in Cycle 2 in any of the three combination therapy arms in study Parts A or B, and no new safety signals were observed. It was determined that olaratumab at 20 mg/kg in combination with doxorubicin, vincristine/irinotecan, or high‐dose ifosfamide, can be safely administered to children with solid or CNS tumors. The frequency and duration of dose delays and modifications were deemed by investigators to be consistent with that experienced in clinical practice with the associated chemotherapy regimens. Serum concentrations of olaratumab were similar to model predictions based on adult exposures. There was no apparent association between Cycle 1 olaratumab exposure by quartile and TEAEs of any grade, or of grade ≥3, further supporting its safety and tolerability. While no trends in data were observed, sample size within categories may have been a limiting factor. No patients developed TEADAs, confirming the low immunogenicity of olaratumab in this population.

The PDGFR pathway has been known to play a role in rhabdomyosarcoma. Studies have demonstrated the PAX3‐FOXO1 fusion oncoprotein, seen in the majority of patients with fusion‐positive alveolar rhabdomyosarcoma, directly increases PDGFRα expression and, in a preclinical model of fusion‐positive alveolar rhabdomyosarcoma, neutralizing antibodies directed against PDGFRα had antitumor activity.^18^, ^19^ In the current trial, definitive conclusions could not be established regarding efficacy due to the limited number of patients in each treatment arm, the three chemotherapy regimens investigated, and the diverse array of tumor types. Nevertheless, the three responses in patients with rhabdomyosarcoma merit further comment, particularly in light of initial data showing activity of olaratumab in combination with doxorubicin in adults with STS and the known role of the PDGFR pathway in rhabdomyosarcoma. There were no clear commonalities among these three patients in terms of treatment arm (ie, Part A/B/C, or chemotherapy regimen), reported histologic subtype, or demographic characteristics (ie, age, sex, geographic region). While olaratumab exposure for each of these patients was above the median, they did not have the highest observed serum concentrations of olaratumab in the study. As concomitant chemotherapy was administered in this trial, the contribution of olaratumab to these responses in patients with rhabdomyosarcoma is unclear. Additionally, tissue samples were not required for participation; therefore, no analysis of PDGFRα status compared to radiographic response was possible (though PDGFRα status has not been shown to correlate with response to olaratumab in other trials).^8^, ^20^ A sustained response in one patient with pineoblastoma is also noteworthy, despite typically poor penetration of monoclonal antibodies (mAbs) across the blood‐brain barrier.^21^ Cerebrospinal fluid (CSF) PK evaluations were not performed in this study, and thus, we were not able to correlate this response to CNS olaratumab concentrations. It is also important to note RANO‐HGG criteria were recommended to evaluate CNS tumors and, since the design of this trial, evaluation criteria have further evolved for CNS tumors in pediatric patients.^22^

Olaratumab at 20 mg/kg was tolerable and safely administered in combination with chemotherapy regimens commonly used in children and adolescents, including doxorubicin, vincristine/irinotecan, or high‐dose ifosfamide. The PK profile of olaratumab in pediatric patients was consistent with the model prediction based on adult data. Variability in olaratumab concentrations was generally higher in Part C, which is largely attributed to the lower number of PK samples available from this study part. It is important to note the PK sampling schedule of this study was purposely developed to be sparse to limit the burden on pediatric patients. Thus, due to limited PK samples, a more formal PK analysis, similar to those in prior adult studies,^8^, ^23^ was not repeated. The observed PK data from this study were overlayed with predictions from the established population PK model which was developed using almost all available PK data from prior studies in adults. This overlay facilitated assessment of the pediatric PK data in the context of the collective understanding of olaratumab PK characteristics. The agreement between the observed PK data with model prediction indicates the PK properties of olaratumab in pediatric patients are similar to those in adults. The olaratumab serum concentrations were higher in pediatric patients with higher body weight, suggesting a potential correlation between PK and age. However, because there is a strong correlation between age and body weight, especially in pediatric subjects, it is difficult to study the correlation between PK and age in isolation of body weight. The PK profile of olaratumab has been extensively studied in adult populations with a wide age range, and no correlation was found between age and PK parameters. A positive correlation was found between body weight and PK in adult subjects, which is common for mAbs.^24^ Although PK properties in adults may not always extrapolate directly to pediatric subjects, for mAb extrapolation, body weight has been found to be the most reliable method.^23^ In pediatrics, age can be an important covariate for PK, especially for drugs that are extensively metabolized or eliminated by mechanisms that have postnatal age‐dependent development.^25^ The elimination pathways of mAbs are not known to show age‐dependent maturation beyond the age limit of most pediatric studies, which is likely the reason why PK of mAbs do not typically correlate with age. Without any modification of the formulation or physical‐chemical structure of olaratumab to enhance blood‐brain barrier penetration, it is very unlikely for CNS concentration of olaratumab to be different from what has been reported for other mAbs, which has consistently been shown to be approximately 0.1–0.2% of steady‐state systemic circulation.^26^ Given our current understanding of the PK properties of olaratumab, and PK of mAbs in general, the correlation of body weight with PK is the most clinically relevant parameter to consider when dosing pediatric patients.

Strengths of this study include rapid accrual and early introduction of combination therapy, and the allowance of investigator choice of chemotherapy. While the only disease type for which olaratumab was being developed at the time of this study was STS, children with primary CNS and non‐sarcoma tumors were eligible, and one patient with a pineoblastoma had clear clinical benefit and received 16 cycles of treatment on study. Despite this strength, the number of diverse tumor types and the assortment of treatment regimens examined did prohibit conclusions regarding olaratumab efficacy in pediatric patients. While no definitive efficacy conclusions could be made, these data may provide insights into other strategies to target PDGFR in pediatric cancers. Assessing half‐life of olaratumab would have been useful but requires three time points per treatment cycle to assess, and only two time points per cycle were available in this study. However, given the similarities reported here between the adult and pediatric PK profiles, the half‐life, estimated as 11 days,^24^ is anticipated to be similar in pediatric and adult patient populations.

The aim of this report is to provide data which benefit future clinical studies of agents targeting the PDGFR pathway in pediatric cancers. The ANNOUNCE phase 3 confirmatory study of olaratumab in combination with doxorubicin in adults with advanced or metastatic STS did not confirm the clinical benefit of olaratumab in combination with doxorubicin as compared to doxorubicin as a standard‐of‐care treatment^20^ and resulted in olaratumab being withdrawn. However, given the potential involvement of the PDGFR pathway in the development of STS and various pediatric malignancies where the PDGFR pathway is implicated,^5^ and the lack of any new first‐line treatments in the last 40 years, the PDGFR pathway remains a potential target for new therapeutics. Additionally, this study demonstrates the feasibility and safety of the addition of mAbs (of a non‐immunomodulatory nature) to standard pediatric chemotherapy regimens, which is critical to advancing combination therapies. Single‐agent treatment with targeted agents that are not directed against oncogenic drivers have limited single‐agent activity and have benefit when combined with cytotoxic chemotherapy or in combination with other targeted agents.^27^, ^28^ The unique design of this clinical trial allowed the successful investigation of targeted monotherapy as a single agent and in combination with cytotoxic chemotherapy agents commonly used in the treatment of childhood and adolescent cancers and serves as a model for accelerated investigation in pediatric oncology to phase 2 and phase 3 clinical trials in relevant disease types.

## 5 CONFLICTS OF INTEREST STATEMENT

L. Mascarenhas reports speaking fees from Bayer and travel expenses from Salarius and Thermo Fisher Scientific. C. Ogawa has no potential conflicts to disclose. T.W. Laetsch reports consulting for Eli Lilly and Company, Loxo Oncology, Bayer, Pfizer, and Novartis, and research funding from Bayer, Pfizer, and Novartis. B.J. Weigel has no potential conflicts to disclose. M.W. Bishop reports research funding from Pfizer. J. Krystal has no potential conflicts to disclose. S.C. Borinstein reports no potential conflicts in the past 12 months and has served on the Advisory Board with Bayer within 2 years. E.K. Slotkin has no potential conflicts to disclose. J.A. Muscal medical advisory board fees from Bayer Pharmaceuticals. P. Hingorani has no potential conflicts to disclose. D.E. Levy has no potential conflicts to disclose. Gary Mo is an employee and stockholder of Eli Lilly and Company and is an inventor on a pending patent application entitled Dosing Regimen. A. Shahir is an employee and stockholder of Eli Lilly and Company. J. Wright is an employee and stockholder of Eli Lilly and Company. S.G. DuBois reports consulting fees and travel expenses from Loxo Oncology prior to acquisition by Eli Lilly and Company, as well as travel expenses from Roche and Salarius.

## ETHICAL APPROVAL

The study protocol was approved by institutional review boards and ethics committees at each participating institution prior to commencing and conducted in accordance with the Declaration of Helsinki.

## Supporting information

Fig S1

Fig S2

Table S1

Table S2

Table S3

Table S4

Table S5

Supplementary Material

## Data Availability

Eli Lilly and Company provides access to all individual participant data collected during the trial, after anonymization, with the exception of pharmacokinetic or genetic data. Data are available to request 6 months after the indication studied has been approved in the United States and EU and after primary publication acceptance, whichever is later. No expiration date of data requests is currently set once data are made available. Access is provided after a proposal has been approved by an independent review committee identified for this purpose and after receipt of a signed data sharing agreement. Data and documents, including the study protocol, statistical analysis plan, clinical study report, blank or annotated case report forms, will be provided in a secure data sharing environment. For details on submitting a request, see the instructions provided at www.vivli.org.
