# Supplementary material for: Phase 1 trial of olaratumab monotherapy and in combination with chemotherapy in pediatric patients with relapsed/refractory solid and central nervous system tumors
Source: Cancer Med. 2021 Jan 20;10(3):843–56. doi: 10.1002/cam4.3658 (PMC7897905; doi:10.1002/cam4.3658)

**Supplementary Figure S1**. Study design (NCT02677116). Part A, olaratumab monotherapy (15 mg/kg) was administered on Day 1 and Day 8 of Cycle 1. Part B, olaratumab monotherapy (20 mg/kg) was administered Day 1 and Day 8 of Cycle 1. If the DLT rate was >33% in Cycle 1 Part A or Cycle 1 Part B the dose was to be reduced. Non-DLT monotherapy patients received combination therapy after Cycle 1. Part C patients received olaratumab (20 mg/kg) in combination with any of the three chemotherapy regimens from Cycle 1. Abbreviations: D, day; DLT, dose-limiting toxicity; *N*, number of patients in group; *n*, number of cycles.


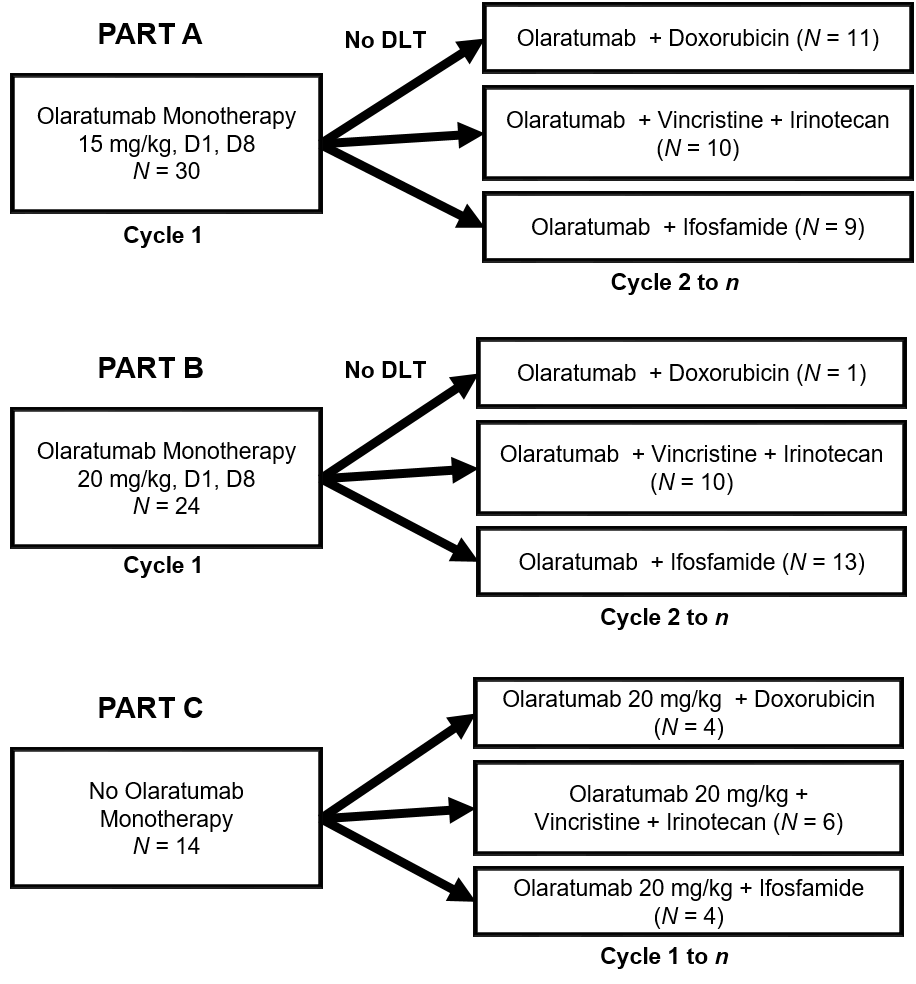

Supplement: Supplementary file 1 — Fig S1 [file CAM4-10-843-s001.docx]
