# Supplementary material for: Phase 1 trial of olaratumab monotherapy and in combination with chemotherapy in pediatric patients with relapsed/refractory solid and central nervous system tumors
Source: Cancer Med. 2021 Jan 20;10(3):843–56. doi: 10.1002/cam4.3658 (PMC7897905; doi:10.1002/cam4.3658)
Supplement: Supplementary file 2 — Fig S2 [file CAM4-10-843-s002.docx]

**Supplementary Figure S2.** Summary of the maximum and minimum olaratumab concentrations following 60-minute intravenous infusions of olaratumab administration on Day 1 and Day 8 of a 21-day cycle. Part A maximum concentrations (Panel A), Part B maximum concentrations (Panel B), Part C maximum concentrations (Panel C), Part A minimum concentrations (Panel D), Part B minimum concentrations (Panel E), and Part C minimum concentrations (Panel F). ^†^Note: Cycle 3-*n* Day 8 represents pooled data from Day 8 for Cycles greater than or equal to 3.

**Panel A.**

**
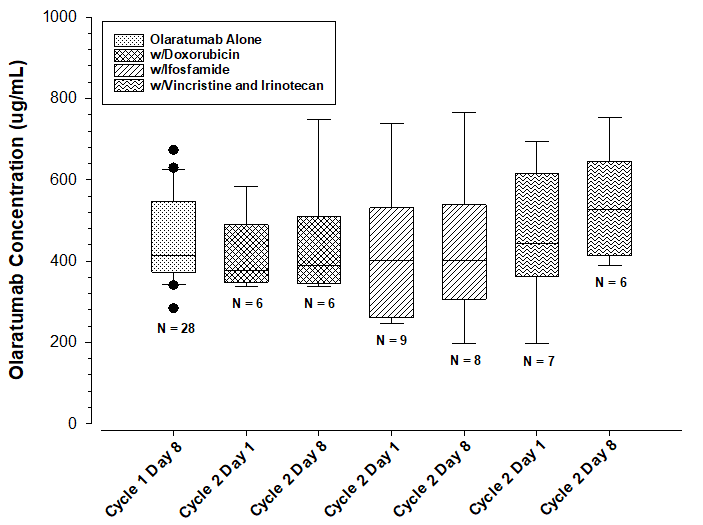
**

**Panel B.**


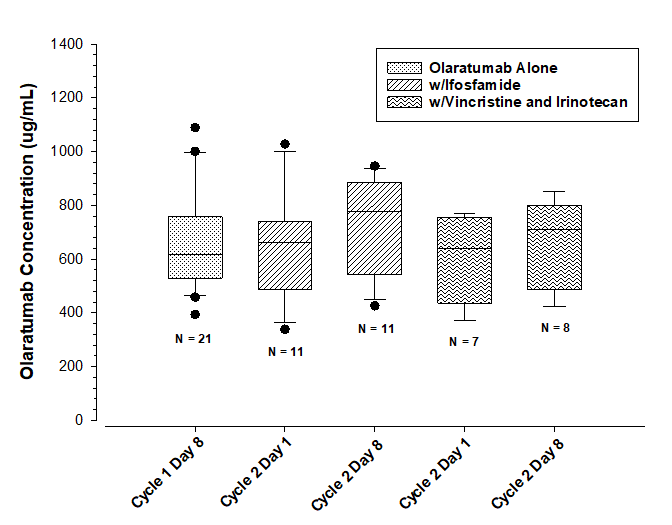


**Panel C.**


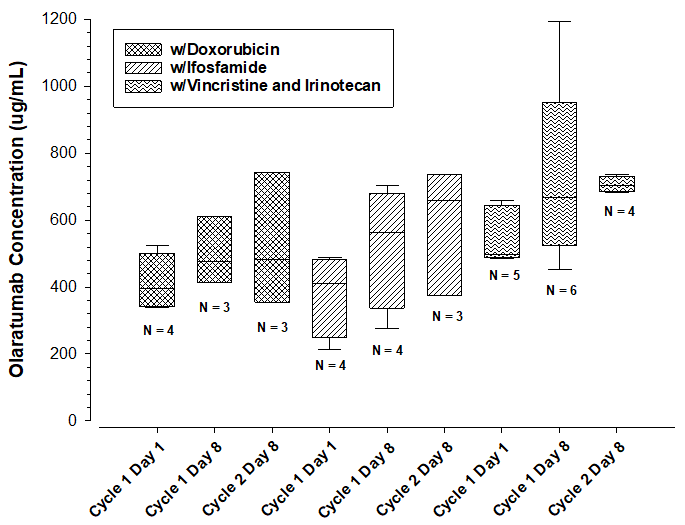


**Panel D.^†^**


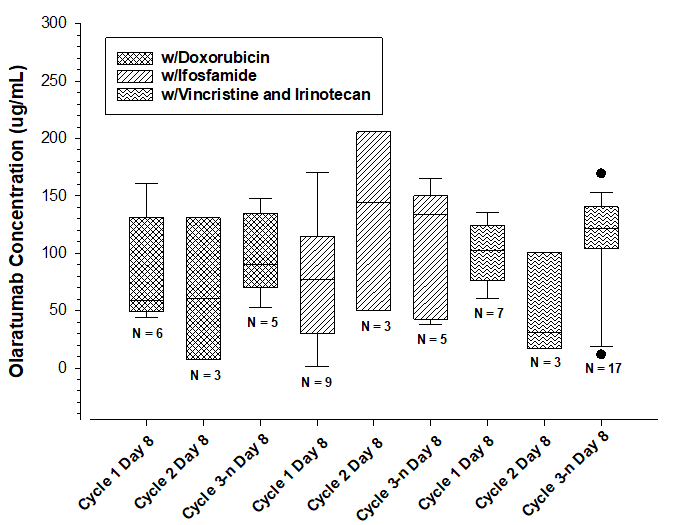


**Panel E.^†^**


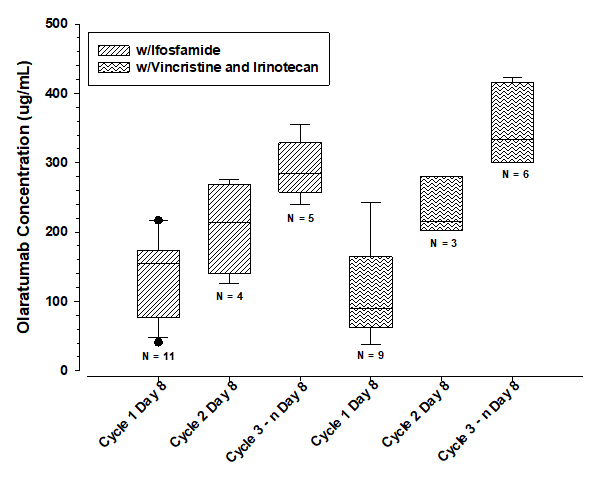


**Panel F.^†^**

**
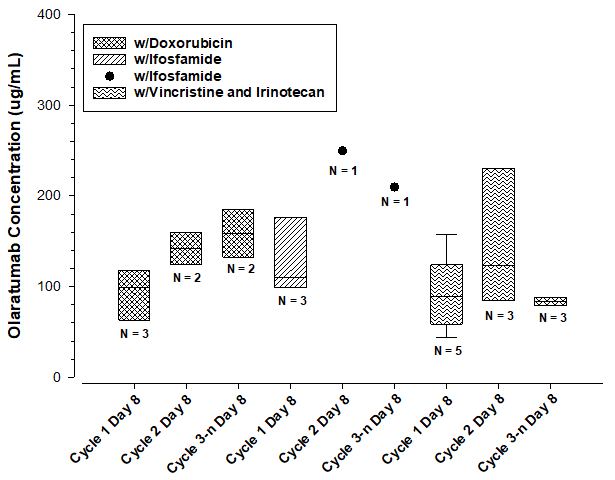
**
