# Supplementary material for: Phase 1 trial of olaratumab monotherapy and in combination with chemotherapy in pediatric patients with relapsed/refractory solid and central nervous system tumors
Source: Cancer Med. 2021 Jan 20;10(3):843–56. doi: 10.1002/cam4.3658 (PMC7897905; doi:10.1002/cam4.3658)
Supplement: Supplementary file 3 — Table S1 [file CAM4-10-843-s003.docx]

**Supplementary Table S1.** Study drug exposure per study drug per study part

|  | **Part A^†^**  ***N* = 30** | | **Part B^‡^**  ***N* = 24** | | **Part C^§^**  ***N* = 14** | |
| --- | --- | --- | --- | --- | --- | --- |
| Study drug | Median treatment duration (weeks) [range] | Median number of cycles | Median treatment duration (weeks)  [range] | Median number of cycles | Median treatment duration (weeks)  [range] | Median number of cycles |
| Olaratumab | 7  [3-78] | 2 | 7  [3-49] | 2 | 13  [4-38] | 4 |
| Vincristine | 10  [3-73] | 3 | 4  [3-46] | 1 | 13  [4-25] | 4 |
| Irinotecan | 9  [3-75] | 3 | 4  [3-46] | 1 | 13  [4-25] | 4 |
| Ifosfamide | 4  [4-19] | 1 | 4  [3-21] | 1 | 13  [4-25] | 4 |
| Doxorubicin | 7  [3-18] | 2 | 0  [N/A] | 0 | 12  [3-18] | 4 |

Abbreviation: *N*, number of patients per study part

^†^Number of patients who received study drug: olaratumab = 30, vincristine/irinotecan = 7, ifosfamide = 9, doxorubicin = 6

^‡^Number of patients who received study drug: olaratumab = 24, vincristine/irinotecan = 9, ifosfamide = 11, doxorubicin = 0

^§^Number of patients who received study drug: olaratumab = 14, vincristine/irinotecan = 6, ifosfamide = 4, doxorubicin = 4
