# Supplementary material for: Phase 1 trial of olaratumab monotherapy and in combination with chemotherapy in pediatric patients with relapsed/refractory solid and central nervous system tumors
Source: Cancer Med. 2021 Jan 20;10(3):843–56. doi: 10.1002/cam4.3658 (PMC7897905; doi:10.1002/cam4.3658)
Supplement: Supplementary file 4 — Table S2 [file CAM4-10-843-s004.docx]

**Supplementary Table S2.** Olaratumab monotherapy treatment-emergent adverse events assessed as related to study drug by the investigator (Cycle 1 only; safety population)

|  | **Part A**  **Olaratumab 15 mg**  ***N* = 30** | | **Part B**  **Olaratumab 20 mg**  ***N* = 24** | |
| --- | --- | --- | --- | --- |
| **System Organ Class**  Preferred Term, *n* (%) | Any Grade | Grade ≥3 | Any Grade | Grade ≥3 |
| **Any adverse event related to study treatment** | 16 (53) | 1 (3) | 15 (63) | 4 (17) |
| **Blood and lymphatic system disorders** |  |  |  |  |
| Anemia | 3 (10) | 0 | 3 (13) | 1 (4) |
| Leukopenia | 0 | 0 | 1 (4) | 0 |
| Lymphopenia | 0 | 0 | 1 (4) | 0 |
| **Cardiac disorders** |  |  |  |  |
| Sinus tachycardia | 0 | 0 | 1 (4) | 0 |
| **Gastrointestinal disorders** |  |  |  |  |
| Nausea | 0 | 0 | 5 (21) | 0 |
| Vomiting | 5 (17) | 0 | 0 | 0 |
| Gastroesophageal reflux disease | 1 (3) | 0 | 0 | 0 |
| Constipation | 1 (3) | 0 | 1 (4) | 0 |
| Abdominal pain | 0 | 0 | 1 (4) | 0 |
| Diarrhea | 0 | 0 | 1 (4) | 0 |
| Stomatitis | 0 | 0 | 1 (4) | 0 |
| **General disorders and administration site conditions** | |  |  |  |
| Fatigue | 3 (10) | 0 | 1 (4) | 0 |
| Pyrexia | 1 (3) | 0 | 0 | 0 |
| Malaise | 0 | 0 | 1 (4) | 0 |
| Early satiety | 0 | 0 | 1 (4) | 0 |
| **Injury, poisoning and procedural complications** | |  |  |  |
| Infusion-related reaction | 0 | 0 | 1 (4) | 0 |
| **Investigations** |  |  |  |  |
| White blood cell count decreased | 3 (10) | 0 | 1 (4) | 0 |
| Alanine aminotransferase increased | 1 (3) | 1 (3) | 1 (4) | 1 (4) |
| Neutrophil count decreased | 1 (3) | 0 | 0 | 0 |
| Platelet count decreased | 2 (7) | 0 | 1 (4) | 0 |
| Aspartate aminotransferase increased | 1 (3) | 1 (3) | 2 (8) | 1 (4) |
| Blood bilirubin increased | 1 (3) | 0 | 0 | 0 |
| Gamma-glutamyl-transferase increased | 0 | 0 | 1 (4) | 1 (4) |
| Blood alkaline phosphatase increased | 0 | 0 | 1 (4) | 0 |
| Blood phosphorus increased | 1 (3) | 0 | 0 | 0 |
| Blood thyroid stimulating hormone increased | 0 | 0 | 1 (4) | 0 |
| Hemoglobin increased | 1 (3) | 0 | 0 | 0 |
| Lymphocyte count decreased | 0 | 0 | 1 (4) | 1 (4) |
| **Metabolism and nutrition disorders** |  |  |  |  |
| Decreased appetite | 0 | 0 | 3 (13) | 0 |
| Hypermagnesemia | 2 (7) | 0 | 0 | 0 |
| Hypoalbuminemia | 0 | 0 | 2 (8) | 0 |
| Hypocalcemia | 2 (7) | 0 | 0 | 0 |
| Hyponatremia | 1 (3) | 0 | 1 (4) | 0 |
| Hyperglycemia | 1 (3) | 0 | 0 | 0 |
| Hyperphosphatemia | 0 | 0 | 1 (4) | 0 |
| Hypophosphatemia | 1 (3) | 0 | 0 | 0 |
| Hypouricemia | 0 | 0 | 1 (4) | 0 |
| **Nervous system disorders** |  |  |  |  |
| Headache | 1 (3) | 0 | 5 (21) | 1 (4) |
| Dysgeusia | 1 (3) | 0 | 0 | 0 |
| **Respiratory, thoracic and mediastinal disorders** | |  |  |  |
| Cough | 0 | 0 | 1 (4) | 0 |
| **Vascular disorders** |  |  |  |  |
| Hypertension | 2 (7) | 0 | 0 | 0 |
| Hypotension | 0 | 0 | 1 (4) | 0 |

Abbreviations: *N*, total number of patients per treatment; *n*, total number of patients per category
