# Supplementary material for: Phase 1 trial of olaratumab monotherapy and in combination with chemotherapy in pediatric patients with relapsed/refractory solid and central nervous system tumors
Source: Cancer Med. 2021 Jan 20;10(3):843–56. doi: 10.1002/cam4.3658 (PMC7897905; doi:10.1002/cam4.3658)
Supplement: Supplementary file 5 — Table S3 [file CAM4-10-843-s005.docx]

**Supplementary Table S3**. Treatment-related treatment-emergent adverse events for all patients for all cycles occurring at grade ≥3 in any treatment arm at any frequency (safety population)

|  | **Part A**  **(*N* = 30)** | | | | | | | | | | | | | | **Part B**  **(*N* = 24)** | | | | | | | | | | | | | | | | **Part C**  **(*N* = 14)** | | | | | | | | | | | | | | |
| --- | --- | --- | --- | --- | --- | --- | --- | --- | --- | --- | --- | --- | --- | --- | --- | --- | --- | --- | --- | --- | --- | --- | --- | --- | --- | --- | --- | --- | --- | --- | --- | --- | --- | --- | --- | --- | --- | --- | --- | --- | --- | --- | --- | --- | --- |
|  | **Olaratumab 15 mg with** | | | | | | | | | | | | | | **Olaratumab 20 mg with** | | | | | | | | | | | | | | | | **Olaratumab 20 mg with** | | | | | | | | | | | | | | |
|  | **Dox *N* = 11** | | | | **Vin/Irin *N* = 10** | | | | | **Ifos *N* = 9** | | | | | **Dox *N* = 1** | | | | | | **Vin/Irin *N* = 10** | | | | | **Ifos *N* = 13** | | | | | **Dox *N* = 4** | | | | | **Vin/Irin *N* = 6** | | | | | **Ifos *N* = 4** | | | | |
| **System organ class** *Preferred term, n (%)* | Any Gr | | Gr≥3 | | | Any Gr | | Gr≥3 | | | Any Gr | | Gr≥3 | | Any Gr | | | Gr≥3 | | | | Any Gr | | Gr≥3 | | | Any Gr | | Gr≥3 | | | Any Gr | | Gr≥3 | | | Any Gr | | Gr≥3 | | | Any Gr | | Gr≥3 | |
| **Blood and lymphatic disorders** | | | | | | | | | | | | | | | | | | | | | | | | | | | | | | | | | | | | | | | | | | | | | |
| Anemia | 4 (36) | | 2 (18) | | | 3 (30) | | 2 (20) | | | 6 (67) | | 5 (56) | | 0 | | | 0 | | | | 4 (40) | | 2 (20) | | | 8 (62) | | 7 (54) | | | 3 (75) | | 2 (50) | | | 3 (50) | | 2 (33) | | | 3 (75) | | 3 (75) | |
| Leukopenia | 0 | | 0 | | | 1 (10) | | 1 (10) | | | 0 | | 0 | | 0 | | | 0 | | | | 2 (20) | | 0 | | | 2 (15) | | 2 (15) | | | 3 (75) | | 3 (75) | | | 3 (50) | | 2 (33) | | | 2 (50) | | 2 (50) | |
| Lymphopenia | 0 | | 0 | | | 1 (10) | | 1 (10) | | | 0 | | 0 | | 0 | | | 0 | | | | 1 (10) | | 1 (10) | | | 2 (15) | | 2 (15) | | | 2 (50) | | 2 (50) | | | 0 | | 0 | | | 2 (50) | | 2 (50) | |
| Neutropenia | 0 | | 0 | | | 1 (10) | | 1 (10) | | | 0 | | 0 | | 0 | | | 0 | | | | 2 (20) | | 2 (20) | | | 2 (15) | | 2 (15) | | | 3 (75) | | 3 (75) | | | 4 (67) | | 4 (67) | | | 2 (50) | | 2 (50) | |
| Thrombocytopenia | 0 | | 0 | | | 1 (10) | | 1 (10) | | | 0 | | 0 | | 0 | | | 0 | | | | 1 (10) | | 0 | | | 3 (23) | | 3 (23) | | | 3 (75) | | 1 (25) | | | 2 (33) | | 0 | | | 0 | | 0 | |
| Febrile neutropenia | 0 | | 0 | | | 0 | | 0 | | | 0 | | 0 | | 0 | | | 0 | | | | 1 (10) | | 1 (10) | | | 7 (54) | | 7 (54) | | | 2 (50) | | 2 (50) | | | 1 (17) | | 1 (17) | | | 3 (75) | | 3 (75) | |
| **Gastrointestinal disorders** | | | | | | | | | | | | | | | | | | | | | | | | | | | | | | | | | | | | | | | | | | | | | |
| Vomiting | 2 (18) | | 0 | | | 5 (50) | | 1 (10) | | | 5 (56) | | 1 (11) | | 0 | | | 0 | | | | 5 (50) | | 1 (10) | | | 6 (46) | | 0 | | | 2 (50) | | 0 | | | 2 (33) | | 0 | | | 2 (50) | | 0 | |
| Diarrhea | 1 (9) | | 0 | | | 5 (50) | | 1 (10) | | | 1 (11) | | 0 | | 0 | | | 0 | | | | 7 (70) | | 2 (20) | | | 0 | | 0 | | | 0 | | 0 | | | 6 (100) | | 1 (17) | | | 2 (50) | | 0 | |
| Nausea | 4 (36) | | 1 (9) | | | 4 (40) | | 0 | | | 3 (33) | | 0 | | 1 (100) | | | 0 | | | | 6 (60) | | 1 (10) | | | 7 (54) | | 2 (15) | | | 3 (75) | | 0 | | | 2 (33) | | 0 | | | 3 (75) | | 0 | |
| Stomatitis | 0 | | 0 | | | 1 (10) | | 0 | | | 1 (11) | | 0 | | 0 | | | 0 | | | | 1 (10) | | 0 | | | 2 (15) | | 1 (8) | | | 1 (25) | | 0 | | | 0 | | 0 | | | 2 (50) | | 0 | |
| **Infections and infestations** | | | | | | | | | | | | | | | | | | | | | | | | | | | | | | | | | | | | | | | | | | | | | |
| Lung infection | 0 | | 0 | | | 1 (10) | | 1 (10) | | | 0 | | 0 | | 0 | | | 0 | | | | 0 | | 0 | | | 0 | | 0 | | | 0 | | 0 | | | 0 | | 0 | | | 0 | | 0 | |
| Penile infection | 0 | | 0 | | | 0 | | 0 | | | 1 (11) | | 1 (11) | | 0 | | | 0 | | | | 0 | | 0 | | | 0 | | 0 | | | 0 | | 0 | | | 0 | | 0 | | | 0 | | 0 | |
| **Investigations** | | | | | | | | | | | | | | | | | | | | | | | | | | | | | | | | | | | | | | | | | | | | | |
| Neutrophil count decreased | | 5 (46) | | 5 (46) | | | 3 (30) | | 2 (20) | | | 4 (44) | | 4 (44) | | 0 | | | 0 | | | | 3 (30) | | 1 (10) | | | 3 (23) | | 2 (15) | | | 1 (25) | | 1 (25) | | | 1 (17) | | 0 | | | 2 (50) | | 1 (25) |
| White blood cell decreased | | 3 (27) | | 3 (27) | | | 2 (20) | | 0 | | | 7 (78) | | 5 (56) | | 0 | | | 0 | | | | 3 (30) | | 1 (10) | | | 4 (31) | | 3 (23) | | | 1 (25) | | 1 (25) | | | 1 (17) | | 1 (17) | | | 2 (50) | | 1 (25) |
| Platelet count decreased | | 3 (27) | | 2 (18) | | | 0 | | 0 | | | 7 (78) | | 6 (67) | | 0 | | | 0 | | | | 1 (10) | | 0 | | | 5 (39) | | 4 (31) | | | 0 | | 0 | | | 3 (50) | | 0 | | | 1 (25) | | 1 (25) |
| Lymphocyte count decreased | | 2 (18) | | 1 (9) | | | 1 (10) | | 0 | | | 5 (56) | | 5 (56) | | 0 | | | 0 | | | | 1 (10) | | 1 (10) | | | 0 | | 0 | | | 0 | | 0 | | | 2 (33) | | 1 (17) | | | 1 (25) | | 1 (25) |
| AST increased | | 1 (9) | | 0 | | | 1 (10) | | 0 | | | 3 (33) | | 1 (11) | | 1 (100) | | | 1 (100) | | | | 1 (10) | | 0 | | | 4 (31) | | 0 | | | 0 | | 0 | | | 2 (33) | | 0 | | | 2 (50) | | 0 |
| GGT increased | | 0 | | 0 | | | 0 | | 0 | | | 4 (44) | | 1 (11) | | 1 (100) | | | 1 (100) | | | | 0 | | 0 | | | 0 | | 0 | | | 0 | | 0 | | | 1 (17) | | 0 | | | 1 (25) | | 0 |
| ALT increased | | 0 | | 0 | | | 1 (10) | | 1 (10) | | | 2 (22) | | 1 (11) | | 1 (100) | | | 1 (100) | | | | 1 (10) | | 0 | | | 2 (15) | | 0 | | | 0 | | 0 | | | 3 (50) | | 1 (17) | | | 2 (50) | | 0 |
| **Metabolism and nutrition disorders** | | | | | | | | | | | | | | | | | | | | | | | | | | | | | | | | | | | | | | | | | | | | | |
| Hypokalemia | | 0 | | 0 | | | 2 (20) | | 0 | | | 1 (11) | | 1 (11) | | | 0 | | | 0 | | | 0 | | 0 | | | 4 (31) | | 3 (23) | | | 0 | | 0 | | | 0 | | 0 | | | 0 | | 0 |
| Hypophosphatemia | | 0 | | 0 | | | 2 (20) | | 0 | | | 2 (22) | | 0 | | | 0 | | | 0 | | | 1 (10) | | 0 | | | 3 (23) | | 1 (8) | | | 0 | | 0 | | | 1 (17) | | 0 | | | 1 (25) | | 0 |
| Dehydration | | 0 | | 0 | | | 1 (10) | | 0 | | | 0 | | 0 | | | 0 | | | 0 | | | 3 (30) | | 1 (10) | | | 0 | | 0 | | | 0 | | 0 | | | 0 | | 0 | | | 0 | | 0 |
| Hyponatremia | | 0 | | 0 | | | 2 (20) | | 0 | | | 0 | | 0 | | | 0 | | | 0 | | | 0 | | 0 | | | 3 (23) | | 1 (8) | | | 0 | | 0 | | | 0 | | 0 | | | 0 | | 0 |
| Hypocalcemia | | 0 | | 0 | | | 2 (20) | | 0 | | | 4 (44) | | 0 | | | 0 | | | 0 | | | 0 | | 0 | | | 2 (15) | | 1 (8) | | | 0 | | 0 | | | 0 | | 0 | | | 0 | | 0 |
| **Nervous system disorders** | | | | | | | | | | | | | | | | | | | | | | | | | | | | | | | | | | | | | | | | | | | | | |
| Headache | | 0 | | 0 | | | 2 (20) | | 0 | | | 2 (22) | | 0 | | | 0 | | | 0 | | | 5 (50) | | 0 | | | 2 (15) | | 1 (8) | | | 2 (50) | | 0 | | | 0 | | 0 | | | 1 (25) | | 0 |
| Peripheral motor neuropathy | | 0 | | 0 | | | 1 (10) | | 1 (10) | | | 0 | | 0 | | | 0 | | | 0 | | | 1 (10) | | 0 | | | 0 | | 0 | | | 0 | | 0 | | | 1 (17) | | 0 | | | 0 | | 0 |
| Seizure | | 0 | | 0 | | | 0 | | 0 | | | 1 (11) | | 1 (11) | | | 0 | | | 0 | | | 0 | | 0 | | | 0 | | 0 | | | 0 | | 0 | | | 0 | | 0 | | | 0 | | 0 |
| **Renal and urinary disorders** | | | | | | | | | | | | | | | | | | | | | | | | | | | | | | | | | | | | | | | | | | | | | |
| Acute kidney injury | | 0 | | 0 | | | 0 | | 0 | | | 0 | | 0 | | 0 | | | 0 | | | | 1 (10) | | 1 (10) | | | 0 | | 0 | | | 0 | | 0 | | | 0 | | 0 | | | 0 | | 0 |
| Fanconi syndrome acquired | | 0 | | 0 | | | 0 | | 0 | | | 0 | | 0 | | 0 | | | 0 | | | | 0 | | 0 | | | 1 (8) | | 1 (8) | | | 0 | | 0 | | | 0 | | 0 | | | 0 | | 0 |

Abbreviations: ALT, alanine aminotransferase; AST, aspartate aminotransferase; Dox, doxorubicin; GGT, gamma-glutamyl transferase; Gr, grade; Ifos, ifosfamide; *N*, number of patients per treatment arm; *n*, number of patients with specified event; Vin/Irin, vincristine/irinotecan.
