# Supplementary material for: Phase 1 trial of olaratumab monotherapy and in combination with chemotherapy in pediatric patients with relapsed/refractory solid and central nervous system tumors
Source: Cancer Med. 2021 Jan 20;10(3):843–56. doi: 10.1002/cam4.3658 (PMC7897905; doi:10.1002/cam4.3658)
Supplement: Supplementary file 6 — Table S4 [file CAM4-10-843-s006.docx]

**Supplementary Table S4.** Serious treatment-emergent adverse events related to study treatment across all study arms for all cycles combined (safety population)

|  | Total (*N* = 68) |
| --- | --- |
| Preferred Term | *n* (%) |
| Subjects with ≥1 SAE related to study treatment | 19 (28) |
| Febrile neutropenia | 11 (16) |
| Anemia | 2 (3) |
| Diarrhea | 2 (3) |
| Neutrophil count decreased | 2 (3) |
| Acute kidney injury | 1 (2) |
| Alanine aminotransferase increased | 1 (2) |
| Aspartate aminotransferase increased | 1 (2) |
| Dehydration | 1 (2) |
| Lung infection | 1 (2) |
| Nausea | 1 (2) |
| Penile infection | 1 (2) |
| Seizure | 1 (2) |
| Vomiting | 1 (2) |

Abbreviations: *N*, number of subjects in analysis population; *n*, number of subjects with a SAE considered to be related to study treatment; SAE, serious adverse event
