# Supplementary material for: Phase 1 trial of olaratumab monotherapy and in combination with chemotherapy in pediatric patients with relapsed/refractory solid and central nervous system tumors
Source: Cancer Med. 2021 Jan 20;10(3):843–56. doi: 10.1002/cam4.3658 (PMC7897905; doi:10.1002/cam4.3658)
Supplement: Supplementary file 7 — Table S5 [file CAM4-10-843-s007.docx]

**Supplementary Table S5.** Breakdown of any grade and grade ≥3 TEAEs, by maximum serum concentration^†^ (Cmax) of olaratumab in Cycle 1

|  | TEAE any grade (grade ≥3) | | | | | | | | |  |
| --- | --- | --- | --- | --- | --- | --- | --- | --- | --- | --- |
|  | Part A | | | Part B | | | Part C | | |  |
|  | Dox  *n* = 11 | Vin/Irin  *n* = 10 | Ifos  *n* = 9 | Dox  *n* = 1 | Vin/Irin  *n* = 10 | Ifos  *n* = 13 | Dox  *n* = 4 | Vin/Irin  *n* = 6 | Ifos  *n* = 4 | |
| <25^th^ percentile | 5 (4) | 4 (3) | 3 (3) |  |  |  | 2 (2) |  | 2 (2) | |
| N | 5 | 4 | 3 |  |  |  | 2 |  | 2 | |
| 25^th^-50^th^ percentile | 3 (2) | 3 (1) | 2 (2) |  |  | 2 (2) | 1 (1) | 2 (2) | 2 (2) | |
| N | 3 | 3 | 2 |  |  | 2 | 1 | 2 | 2 | |
| 50^th^-75^th^ percentile | 1 (1) | 2 (2) | 1 (1) |  | 6 (4) | 3 (2) | 1 (1) | 1 (1) |  | |
| N | 1 | 2 | 1 |  | 6 | 3 | 1 | 1 |  | |
| ≥75^th^ percentile |  | 1 (1) | 3 (3) |  | 2 (2) | 8 (6) |  | 2 (2) |  | |
| N |  | 1 | 3 |  | 2 | 8 |  | 2 |  | |
| Total | 9 | 10 | 9 | 0 | 8 | 13 | 4 | 5 | 4 | |

Abbreviations: Dox, doxorubicin; Ifos, ifosfamide; *N*, number of patients per treatment arm; *n*, number of patients with specified event; TEAE, treatment-emergent adverse event; Vin/Irin, vincristine/irinotecan.

^†^Olaratumab maximum serum concentration quartiles were calculated across all doses.
