# Supplementary material for: Phase 1 trial of olaratumab monotherapy and in combination with chemotherapy in pediatric patients with relapsed/refractory solid and central nervous system tumors
Source: Cancer Med. 2021 Jan 20;10(3):843–56. doi: 10.1002/cam4.3658 (PMC7897905; doi:10.1002/cam4.3658)
Supplement: Supplementary file 8 — Supplementary Material [file CAM4-10-843-s008.docx]

**Supporting Information**

**Supplementary Methods.** Pharmacokinetic parameter estimates of adult population.

| **Parameter description** | **Population estimate  (%SEE)** | **Inter-patient variability (%SEE)** |
| --- | --- | --- |
| **Structural model** |  |  |
| Clearance, CL (L/h) | 0.0193 (1.8) | 36.3% (9.8) |
| Central volume of distribution, V_1_ (L) | 3.54 (1.2) | 26.1% (21.0) |
| Peripheral volume of distribution, V_2_ (L) | 2.18 (8.6) |  |
| Inter-compartmental clearance rate, Q (L/h) | 0.0405 (16.1) |  |
|  |  |  |
| **Covariate effects** |  |  |
| WTE_CL_^†^ | 0.495 (7.9) |  |
| WTE_V1_^‡^ | 0.620 (8.8) |  |
| TUMR_CL_^†^ | 0.00160 (16.1) |  |
|  | | |
| **Residual error** |  | |
| Additive (µg/mL) | 11.1 (18.5) | |
| Proportional | 25.3% (5.6) | |

Abbreviations: SEE, standard error of the estimate; TUMR_CL_, tumor size effect on clearance; WTE_CL_, body weight effect on clearance; WTE_V1_, body weight effect on central volume of distribution.

^†^CL_ind_, CL * (WTE/median(WTE))^WTE_CL_ * (1 + TUMR_CL_ * (TUMR – median(TUMR)).

^‡^V_1ind_, V_1_ * (WTE/median(WTE))^WTE_V1_.

**Supplementary Methods Legend.** Body weight of the simulation trial population was selected to reflect that of the study population. The simulation overlay was separated into two groups based on the median body weight of 35.8 kg. The simulation, thus, included two simulated trial populations:

- - 100 pediatric patients with uniform distribution of body weight from 11-36 kg
  - 100 pediatric patients with uniform distribution of body weight from 36.1-78 kg

The same population was used to simulate both the 15 mg/kg olaratumab treatment and 20 mg/kg treatment.

Model parameters were allometrically scaled to pediatric patients using the following equations:

$$CL_{ped}=CL_{adult}\cdot\left( \frac{WT_{ped}}{WT_{adult}} \right)^{0.75}$$

$$Q_{ped}=Q_{adult}\cdot\left( \frac{WT_{ped}}{WT_{adult}} \right)^{0.75}$$

$${V_{1}}_{ped}={V_{1}}_{adult}\cdot\left( \frac{WT_{ped}}{WT_{adult}} \right)^{1}$$

$${V_{2}}_{ped}={V_{2}}_{adult}\cdot\left( \frac{WT_{ped}}{WT_{adult}} \right)^{1}$$
